# Stoichiometry patterns in the androdioecious *Acer tegmentosum*

**DOI:** 10.1038/srep35022

**Published:** 2016-10-11

**Authors:** Xinna Zhang, Jie Yao, Chunyu Fan, Lingzhao Tan, Chunyu Zhang, Juan Wang, Xiuhai Zhao, Klaus von Gadow

**Affiliations:** 1 Key Laboratory for Forest Resources & Ecosystem Processes of Beijing, Beijing Forestry University, Beijing 100083, China; 2 Faculty of Forestry and Forest Ecology, Georg-August-University Göttingen, Büsgenweg 5, D-37077 Göttingen, Germany; 3 Department of Forest and Wood Science, University of Stellenbosch, South Africa

## Abstract

This study evaluates stoichiometry patterns in the androdioecious *Acer tegmentosum*, a species characterized by a rare reproductive system where males and hermaphrodites coexist. Altogether 31 hermaphrodites and 29 male plants were harvested and samples of leaves, current-year shoots, branches and coarse roots were analyzed to explore gender differences in biomass, C, N and P concentrations of these four components. The nitrogen to phosphorus relationship of each component was examined using SMA estimates. Males had significantly greater amounts of leaf and coarse root dry matter content than hermaphrodites. C, N and P stoichiometry differed significantly between genders, especially in the newly emerging vegetative components (leaves and shoots). Males had higher C/N and C/P ratios in current-year shoots and lower C/P ratios in leaves and branches. Hermaphrodites had higher N/P ratios in the leaves and branches. Males had higher rates of increase in leaf P content than hermaphrodites. This study suggests that stoichiometry patterns may be significantly affected by gender.

Androdioecy is a rare sexual dimorphic reproductive system, characterized by the co-existence of hermaphrodites and males in the same reproductive population^1^. Investigations into evolutionary and maintenance mechanism revealed that most of these species turned out to be functional dioecious species^2^,^3^,^4^. Morphologically, the flowers of the hermaphrodite individuals of an androdioecious species act like female flowers, because they fail to produce fertile pollen.

Some disagreement still exists regarding the role of sexual dimorphism in resource allocation between genders, which was thought to lead to sexual differences in nutrient consumption and in the reproduction functions between males and females^5^,^6^,^7^,^8^. A number of studies examined gender differences in resource allocation, but some doubt still remains regarding the trade-off patterns in females. In most investigations it is assumed that the higher reproductive allocation is associated with some cost, such as reduced rates of growth^7^,^9^,^10^,^11^ and higher mortality rates^12^,^13^. Hermaphrodites have to allocate their limited energy not only to flowers but also to the production of seeds and fruits, while males invest into flowers only. Contrary to expectations, it was found that females have specific mechanisms which may be compensatory, alleviating the higher reproductive effort^7^,^14^,^15^.

Differences in resource allocation between genders to reproductive and vegetative growth may contribute to differences in nutrient consumption. Comparative tests of sexual differences have come mostly from studies of resource allocation patterns in dioecious plants. Little attention has been paid to stoichiometry patterns, even less when such patterns refer to androdioecious species. Biomass and nitrogen distribution, which reveal the resources allocation pattern of different sexes in dioecious species is particularly important in studies about reproductive costs^16^. Gender-related differences in the concentrations of the primary elements, C, N and P would advance understanding of resource allocation patterns^17^. Carbon is the main element constituting the dry matter components of plants^18^. If males have a higher growth rate than hypothesized, higher carbon content would be found in male components when compared with those in hermaphrodites. The growth metabolism of plants requires proteins and nucleic^19^,^20^. Therefore, nitrogen and phosphorus are also important when comparing growth rates between genders. Of particular interest are the differences in the relationship between nitrogen and phosphorus which are essential elements for photosynthetic capacity and growth^21^. The differences in the gender-related reproductive cost suggest that individual trees vary in their nutrient content and its distribution.

This study explores the C:N:P stoichiometry between the genders of the androdioecious *Acer tegmentosum*. Gender related differences are expected to be revealed in the nutrient concentrations of leaves, current-year shoots, branches and coarse roots. In particular interest are the relationships between nitrogen and phosphorus concentrations in hermaphrodite and male trees. Accordingly, the objectives of this study were (1): to examine whether the C, N, P concentrations in the four different plant components vary between genders and (2) to evaluate differences in the nitrogen-phosphorus concentration relationships between the genders.

## Results

### Component amounts of dry matter content of both genders

The amounts of dry matter content of fruits (only for hermaphrodites), leaves, current-year shoots, branches and coarse roots from both male and hermaphrodite trees were first analyzed. The results show that neither the amount of the current-year shoots dry matter content, nor that of branches differed between sexes (Table 1). The amount of dry matter content of the leaves and coarse roots of males were slightly higher than those of the hermaphrodites (*p* < 0.05).

### Biomass components as a proportion and C, N and P content

**Figure 1:**
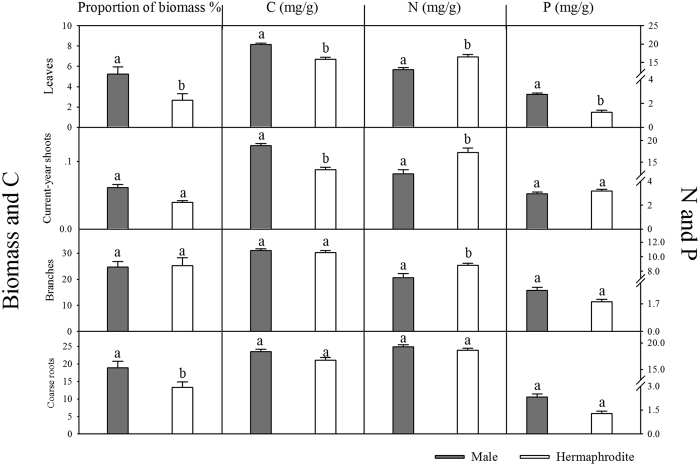
Comparison of biomass proportion and C, N and P concentrations of the four components in hermaphrodite and male trees. Different letters within each plot indicate significant differences (*p* < 0.01) between genders, in an ANCOVA model with *DBH* and gender as covariates.

The four biomass components of hermaphrodites and males were determined as a proportion of the total biomass. Compared with males, hermaphrodites had a lower ratio of leaf and coarse root biomass. This result suggests that hermaphrodites have a different biomass allocation strategy than males. In terms of element content, males had higher amounts of C in the newly emerging vegetative components (leaves and shoots) than hermaphrodites and a higher P content in the leaves. However, hermaphrodites contain higher amounts of N than males, in the leaves, in current-year shoots and in branches. The element amounts in the coarse roots were not significantly different between the sexes. The results are shown in Fig. 1.

### Differences of stoichiometric ratios in both genders

As shown in Table 2, mean C/N, C/P and N/P ratios in all components are gender-specific (based on the results of *t*-tests), save for coarse roots and the N/P ratio in current-year shoots. A closer look at the data shows that males have higher C/N ratios in leaves, current-year shoots and branches as well as a higher C/P ratio in current-year shoots, but lower C/P ratios in leaves and branches. The N/P ratios differed significantly between the sexes, with higher concentrations in leaves and branches of hermaphrodites than in males.

### Gender-related nitrogen-phosphorus relationships

**Figure 2:**
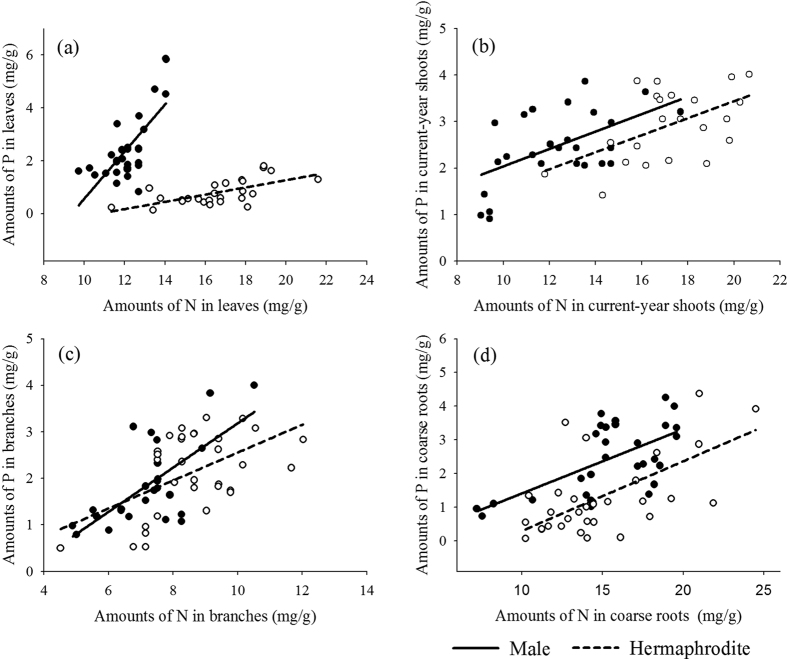
Phosphorus concentrations as a function of the amount of nitrogen in four components of hermaphrodite and male trees. Details of these relationships, calculated from standardized major axis (SM) regressions, are presented in Table 3.

The nitrogen-phosphorus relationships were also analyzed for the genders and among their SMA component estimates. Figure 2 and Table 3 show for all the observed components that the P concentration increased with an increase in N concentration. However, both the regression intercepts and the slopes vary between male and hermaphrodite trees. The data for current-year shoots, branches and coarse roots show that the scales of phosphorus concentrations with respect to nitrogen concentrations differ between the two gender groups although in both the slope < 1.0.

This result shows that increases in the amount of P fail to keep pace with increasing N concentrations (Fig. 2b–d and Table 3). The SMA regression slope of leaves in males exceeds 1, which is statistically significant, while the slope in hermaphrodites is less than 1. The OLS regression shows the same result of non-overlapping slopes (Table 4). The results of these two tests suggests that the amount of P in the leaves of males accelerates faster than a unit increase in the amount of N in leaves, i.e., the rate of increase in the amount of P in male trees is greater than that of hermaphrodites (Tables 3 and 4).

## Discussion

### Sex-differential biomass allocation and dry matter content

Earlier studies on sexual dimorphism revealed different resource allocation patterns between female and male trees^11^,^12^,^13^. It was found that females usually had a greater energy demand during reproduction. Higher reproductive costs affected the vegetative growth of female plants, resulting in different growth rates and biomass allocation patterns^7^,^22^,^23^,^24^. However, in many cases no differences were found between the genders in resource allocation^8^,^25^,^26^.

Our analysis of the contribution to total biomass of the four vegetative structures revealed significant differences between the genders, hermaphrodites having lower ratios of leaf to coarse root biomass than males (Fig. 1). This suggests that *A. tegmentosum* has a gender-specific biomass allocation strategy, with males allocating more resources to leaves and coarse roots.

The reproductive investment can be measured by the dry matter content of its reproductive and vegetative parts^27^. The fact that some potentially limiting resource, such as carbon, is not evenly distributed between the different plant organs is often ignored^28^. Biomass as well as energy can pass between vegetative and reproductive organs^29^. The amounts of dry matter content can reflect the nutrient-holding and resource-retention capacity of plants^30^,^31^. The amount of dry matter content of leaves is a leaf trait, easily measured and often reported. Díaz-Barradas suggested that female plants of the dioecious *Empetrum rubrum* species have greater amounts of leaf dry matter content and may exhibit compensatory mechanisms to face their higher reproductive costs^32^. It has also been reported that males have lower amounts of dry matter content but higher levels of protein^33^. In this study, higher amounts of leaf and coarse root dry matter content were found in males than in hermaphrodites in *A. tegmentosum* (Table 1), suggesting that males have a greater ability of acquiring and retaining carbon in leaves and coarse roots.

### Differential C, N and P concentration between genders

For sexually dimorphic species, the growth rate of female and male plants were found to be significantly different, because of the differences in reproductive investment between them^15^. The indispensable elements C, N and P have a bearing on the regulation of organismal growth rates^34^. But only a few studies deal with the nutrient concentrations in relation to the growth rates, and most of these studies were carried out under controlled conditions. Gehring (1994), analysed the sexual differences of growing individual male and female plants of the dioecious species *Silene latifolia* under specific conditions and found that higher female reproductive costs did not significantly affect their ability to assimilate carbon^35^. Che-Castaldo (2015) demonstrated that female plants allocate more resources (in terms of carbon, nitrogen and phosphorus) to reproduction than males^36^. Similarly, Wallace (1979) reported that male plants allocate a greater proportion of their resources (including biomass, nitrogen and phosphorus) to reproductive tissues relative to female plants^37^. Zhang *et al*.^11^ suggested that the strategies to cope with N and P deficiencies is different between the sexes and that deficiencies in the amounts of N and P would have greater negative effects on females than on males^38^. The amount of nitrogen is one of the most important factors affecting photosynthetic efficiency, especially nitrogen in the leaves which is mostly allocated to the photosynthetic organs^39^,^40^. Photosynthetic efficiency differs among different dioecious species. *Simmondsia chinensis*, *Silene latifolia* and *Populus tremuloides* are reported to have a higher photosynthetic capacity in males than in females, but contrary results were found in *Acer negundo* and *Simmondsia chinensis*^35^,^37^,^41^,^42^,^43^,^44^ Tolvanen (2001) studied the dioecious dwarf shrub *Salix arctica* and reported that males appeared to have lower amounts of N in current shoots and leaves in the early season, and that females were able to allocate more C and N to plant growth^45^.

Figure 1 shows that males have higher levels of C in their new vegetative components (leaves and current-year shoots) than hermaphrodites. Males also have higher P concentrations in their leaves than hermaphrodites. P content is thought to be related to the P concentration in the rRNA, which influences the growth rate. Our results show that, compared with hermaphrodites, males allocated more C and P to vegetative growth. Regarding N concentrations, our results are similar with those of Tolvanen (2001). We also found that hermaphrodites have higher N concentrations in their leaves, current-year shoots and branches (Fig. 1) than males.

During the reproductive season, females tend to adjust their resource allocation patterns by increasing photosynthesis to meet the demands of flowers or fruits^14^,^42^. In this study, the samples were collected in the fruiting season, which may explain the higher N content in hermaphrodites than in males, owing to the C demands in fruits. Our results show a clear, gender-differential pattern of nutrient content. This supports the hypothesis that sexual dimorphic traits have evolved to meet the different resource and nutrient requirements for males and hermaphrodites.

### Gender-related stoichiometric ratios and the nitrogen-phosphorus relationship

C, N and P make important contributions to the formation of carbohydrates, lignin, cellulose, proteins and other substances associated with the regulation of growth rates, which are especially affected by N and P^20^. High phosphorous levels may result in lower C/P and N/P ratios^46^. Usually, the N/P ratio in leaves accurately reflects the dynamic characteristic of the nutrient status^47^. This ratio has therefore been used as a diagnostic indicator of the limitation of vegetative growth by these nutrients^47^,^48^. The C:N:P ratios of organisms are mainly determined by their P content^49^. The growth rate hypothesis (GRH) states that plants themselves will change their specific organ C:N:P ratios to adapt to the change of growth rates. Therefore, the organisms with higher growth rates exhibit relatively lower C:P and N:P ratios^20^.

For sexually dimorphic species, the higher reproductive investment may affect the growth rate of females such that male plants usually have higher vegetative growth rates^12^,^15^,^50^. According to that growth rate hypothesis, the sexually differential growth rates may be reflected in the C:N:P ratios of particular plant components between males and hermaphrodites. A limitation of specific nutritional elements may transform the chemical constitution of individual plants, contributing to changes in the stoichiometric ratios. As a result, the C/N, C/P and N/P ratios are decreasing with increasing growth rates^51^. When the P concentration in plant cells increases erratically, the growth rate increases but the N/P ratio drops^20^,^52^.

As shown in Table 2, hermaphrodites have a lower C/N ratio in leaves, current-year shoots and branches, as well as a lower C/P ratio in current-year shoots, but a higher C/P ratio in leaves and branches. In terms of N/P ratios, males show lower ratios in leaves and branches, suggesting that males may have higher rates of growth in leaves and branches than hermaphrodites.

We also analyzed the nitrogen-phosphorus relationships of the four components. The results of slopes based on SMA and OLS regressions were consistent, showing different intercepts and slopes between genders (Tables 3 and 4). The P concentration increased with increasing N in both sexes, but the slope of the regression of male leaves exceeds 1, while it is less than 1 in hermaphrodites. The scaling slope of leaves was greater in males than in hermaphrodites, suggesting that leaf P increased faster relative to leaf N in males and that males have a greater ability to absorb phosphorus.

These new findings on the androdioecious *A. tegmentosum* may hopefully lead to comparative studies on stoichiometry patterns of other sexually dimorphic species, especially regarding the different reproductive and vegetative components.

## Methods

### Study region

The original experiments were conducted at the Jiaohe Experimental Forest in Jilin Province (43°57.5′N, 127°44.1′–127°44.7′E) with a monsoon climate, at an average elevation of 459 m. The region has a mean annual temperature of 3.8 ^o^C, precipitation is low in winter but at higher elevations very high in the summer, with a mean annual precipitation as high as 695.9 mm. Located in northeastern China, the study site is a multi-species near-mature forest dominated by *Pinus koraiensis*, *Fraxinus mandshurica*, *Tilia amurendsis* and *Acer mono*. The soil is a dark brown forest soil.

The androdioecious species *A. tegmentosum* in this study is a small tree widely distributed in the conifer and broad-leaved mixed forests in northeastern China, which blossoms in early spring with racemes of green flowers. The fruits of the hermaphrodite plants emerge with a dipterous appearance and change from green to yellowish-brown when ripe in late August.

### Sampling in the field

We randomly identified the sex of 60 flowering *A. tegmentosum* (29 males and 31 hermaphrodites) in the spring of 2014 and marked their flowering branches. All the sample trees were cut at ground level in order to harvest their leaves and shoots, as well as the fruits from the hermaphrodites. The flowering branches were dissected into leaves, current-year shoots and branches. The coarse roots (diameter ≥ 5 mm) of each tree were dug out and cleaned. The fresh mass of each component was determined separately in the field using a platform balance to the nearest 0.1 kg. Representative samples of each of the four components, weighing between 500 and 1000 g were taken to the laboratory for analysis.

### Measurements of biomass components and element analysis

After measuring the fresh weights, the samples collected in the field were dried in an oven to a constant mass and weighed. To calculate the total biomass (expressed as dry matter) of each section, we multiplied the ratio of samples (dry to fresh matter) by total fresh weight as measured in the field^53^. Proportion of biomass was defined as the ratio of biomass of the corresponding components to total biomass. The sample dry matter content was measured by the ratio of dry mass to fresh mass (g/kg)^54^.

Before the element analysis, the dried samples were ground into a powder. The C concentrations were determined from approximately 100 mg of homogenously ground material of each sample, using a potassium dichromate volumetric method^55^. The total amount of N of each sample was determined by the Kjeldahl Method and P by the Mo-Sb colorimetric method^55^.

### Data analysis

Analyses of covariance (ANCOVA) with diameter at breast height (*DBH*) and gender as covariates were applied to examine sexual differences in biomass proportion and the C, N and P concentrations of the various components. Standardized major axes estimation (SMA) is a procedure for assessing the heterogeneity of regression slopes, to identify the best fit bivariate line between two variables^56^,^57^. The most common use of the null hypothesis is that the slopes are equal to one, which implies isometry, with *y* and *x* increasing at a constant rate^58^. This method was used to fit a regression line for the N concentrations against the P concentrations, and then the slopes of these lines were compared between genders. If the slopes significantly differed from 1, the relationship between N against P concentrations would not be described as isometric. Measurement error (ME) is the difference between the measured values and actual values, which can cause a biased estimate of the slopes in linear regression^58^. McArdle (2003) pointed out that one efficient way to deal with ME is to test the slopes of both *y* on *x* and *x* on *y*^59^. In this study, the ordinary least squares (OLS) regression was also used to analyze the nitrogen-phosphorus relationship in the four components between genders. We regressed both N against P concentration and P against N concentration to deal with the ME in the variables.

All analyses were performed by R, version 3.1.3; the R library “smatr” was used for SMA analyses.

## Additional Information

**How to cite this article**: Zhang, X. *et al*. Stoichiometry patterns in the androdioecious *Acer tegmentosum. Sci. Rep.* **6**, 35022; doi: 10.1038/srep35022 (2016).

## Figures and Tables

**Table 1:** Amounts of dry matter content of various components in both genders.

Dry matter content (g/kg)	Male	Hermaphrodite	*p*-value
Fruit dry matter content	—	280.79 ± 8.71	—
Leaf dry matter content	254.53 ± 0.72	234.65 ± 1.57	*
Current-year shoot dry matter content	432.02 ± 27.97	400.29 ± 3.04	n.s.
Branch dry matter content	468.28 ± 0.99	461.98 ± 0.71	n.s.
Coarse root dry matter content	466.93 ± 1.92	441.96 ± 1.25	*

Data show means ± SE; *significant at 0.05 level; n.s., not significant.

**Table 2:** C/N, C/P and N/P ratios of the various components on a mass basis.

Items	Components	Genders		*p*-value
		Males	Hermaphrodites	
C/N	Fruits	—	33.41 ± 2.13	—
	Leaves	47.17 ± 0.21	27.83 ± 0.30	***
	Current-year Shoot	44.51 ± 0.45	22.70 ± 0.48	***
	Branches	82.83 ± 0.82	65.73 ± 0.64	**
	Coarse roots	30.83 ± 0.39	28.58 ± 0.33	n.s.
C/P	Fruits	—	245.20 ± 14.44	—
	Leaves	603.83 ± 17.03	875.33 ± 25.90	***
	Current-year Shoot	223.55 ± 4.38	141.18 ± 4.39	***
	Branches	335.27 ± 7.60	496.00 ± 10.75	**
	Coarse roots	776.25 ± 32.89	882.29 ± 38.78	n.s.
N/P	Fruits	—	10.78 ± 0.83	–
	Leaves	12.96 ± 0.36	32.01 ± 0.90	***
	Current-year Shoot	5.15 ± 0.08	6.87 ± 0.29	n.s.
	Branches	4.29 ± 0.11	8.01 ± 0.20	**
	Coarse roots	22.57 ± 0.81	29.98 ± 1.22	n.s.

Results show means ± SE; **significant at 0.01 level; ***significant at 0.001 level; n.s., not significant.

**Table 3:** Summary of standardized major axis (SMA) regression results for the components examined in both genders.

Components	n	Intercept	95% CI		Slope	95% CI		R^2^	*p*-value
			Lower	Upper		Lower	Upper		
Male trees									
Leaves	29	−12.69	−16.90	−8.47	1.25**	0.95	1.64	0.51	*p* < 0.001
Current-year shoots	29	−1.81	−3.26	−0.35	0.35***	0.25	0.48	0.29	*p* < 0.01
Branches	29	−3.19	−4.81	−1.56	0.70**	0.51	0.95	0.46	*p* < 0.001
Coarse roots	29	−2.22	−3.69	−0.75	0.30***	0.22	0.41	0.40	*p* < 0.001
Hermaphrodite trees									
Fruits	31	−2.43	−4.25	−0.61	0.31***	0.21	0.45	0.22	*p* < 0.05
Leaves	31	−2.76	−3.93	−1.60	0.21***	0.16	0.30	0.41	*p* < 0.001
Current-year shoots	31	−3.05	−5.45	−0.65	0.35***	0.24	0.51	0.28	*p* < 0.05
Branches	31	−2.83	−4.44	−1.21	0.58**	0.42	0.79	0.27	*p* < 0.01
Coarse roots	31	−3.72	−5.26	−2.18	0.34***	0.25	0.45	0.37	*p* < 0.001

*Significant deviation of slope from 1, **p < 0.01, ***p < 0.001.

**Table 4:** Data range of slopes using OLS regression (test both N against P concentration and 1/[P against N concentration]) in various components in both genders.

Components	Males		Hermaphrodites	
	Range	*p*-value	Range	*p*-value
Fruits	—	—	0.15–0.65	*p* < 0.001
Leaves	0.89–1.75	*p* < 0.01	0.14–0.34	*p* < 0.001
Current-year shoots	0.19–0.65	*p* < 0.001	0.18–0.66	*p* < 0.001
Branches	0.48–1.03	*p* < 0.001	0.30–1.11	*p* < 0.001
Coarse roots	0.19–0.48	*p* < 0.001	0.21–0.55	*p* < 0.001

*p*-value shows the significant deviation of slope from one.

## References

[b1] Yampolsky C. & Yampolsky H. Distribution of sex forms in the phanerogamic flora. Bibliotheca genetica (Leipzig, 1922).

[b2] Charlesworth D. Androdioecy and the evolution of dioecy. Biol J Linn Soc. 22(4), 333–348 (1984).

[b3] Anderson G. J. & Symon D. E. Functional dioecy and andromonoecy in Solanum. Evolution. 43, 204–219 (1989). 10.1111/j.1558-5646.1989.tb04218.x 28568500

[b4] Vassiliadis C., Valero M. & Saumitou L. P. A model for the evolution of high frequencies of males in an androdioecious plant based on a cross-compatibility advantage of males. Heredity. 85, 413–422 (2000). 11122419 10.1046/j.1365-2540.2000.00755.x

[b5] Delph L. F. Sexual dimorphism resource allocation patterns in the subdioecious shrub *Hebe subalpin*a. Ecology. 71, 1342–1351 (1990).

[b6] Roff D. A. The evolution of life histories: theory and analysis, 1^st^ edn. Chapman and Hall (New York, 1992).

[b7] Obeso J. R. The costs of reproduction in plants. New Phytol. 155, 321–348 (2002). 10.1046/j.1469-8137.2002.00477.x 33873312

[b8] Petzold A., Pfeiffer T., Jansen F., Eusemann P. & Schnittler M. Sex ratios and clonal growth in dioecious Populus euphratica Oliv., Xinjiang Prov., Western China. Trees. 27, 729–744 (2013).

[b9] Antos J. A. & Allen G. A. A comparison of reproductive effort in the dioecious shrub Oemleria cerasiformis using nitrogen, energy and biomass, as currencies. Am Midl Nat. 124, 254–262 (1990).

[b10] Cornelissen T. & Stiling P. Sex-biased herbivory: a meta-analysis of the effects of gender on plant-herbivore interactions. Oikos. 111, 488–500 (2005).

[b11] Zhang X. N., Zhang C. Y. & Zhao X. H. Effect of sex ratio, habitat factors and neighborhood competition on stem growth in the dioecious tree *Fraxinus mandshurica*. Ecol Res. 29, 309–317 (2014).

[b12] Allen G. A. & Antos J. A. Sex ratio variation in the dioecious shrub Oemleria cerasiformis. Am Nat. 141, 537–553 (1993). 19425998 10.1086/285490

[b13] Nanami S., Kawaguchi H. & Yamakura T. Sex ratio and gender dependent neighboring effects in Podocarpus nagi, a dioecious tree. Plant Ecol. 177, 209–222 (2005).

[b14] Tozawa M., Ueno N. & Seiwa K. Compensatory mechanisms for reproductive costs in the dioecious tree Salix integra. Botany. 87, 315–323 (2009).

[b15] Àlvarez-Cansino L., Zunzunegui M., Diaz Barradas M. C. & Paz Esquivias M. Gender-specific costs of reproduction on vegetative growth and physiological performance in the dioecious shrub Corema album. Ann Bot. 106, 989–998 (2010). 20884627 10.1093/aob/mcq197 PMC2990667

[b16] Queenborough S. A., Burslem D. F., Garwood N. C. & Valencia R. Determinants of biased sex ratios and inter-sex costs of reproduction in dioecious tropical forest trees. Am J Bot. 94(1), 67–78 (2007). 21642209 10.3732/ajb.94.1.67

[b17] Sterner R. W. Elemental stoichiometry of species in ecosystems. In Linking Species & Ecosystems. Springer: US, 240–252 (1995).

[b18] Etherington J. R. Plant physiological ecology. Edward Arnold Ltd, 11–16 (1978).

[b19] Aerts R. & Chapin F. S. The mineral nutriation of wild Plants revised:a re-evaluation of processes and patterns. Ecol Res. 30, l–67 (2000).

[b20] Elser J. J., Acharya K. & Kyle M. Growth rate stoichiometry couplings in diverse biota. Ecol Lett. 6, 936–943 (2003).

[b21] Reich P. B., Oleksyn J., Wright I. J., Niklas K. J., Hedin L. & Elser J. J. Evidence of a general 2/3-power law of scaling leaf nitrogen to phosphorus among major plant groups and biomes. P Roy Soc Lond B Bio. **277(1683)**, 877–883 (2010). 10.1098/rspb.2009.1818 PMC2842731 19906667

[b22] Obeso J. R., Alvarez S. M. & Retuerto R. Sex ratios, size distributions, and sexual dimorphism in the dioecious tree Ilex aquifolium (Aquifoliaceae). Am J Bot. 85, 1602–1608 (1998). 21680320

[b23] Leigh A., Cosgrove M. J. & Nicotra A. B. Reproductive allocation in a gender dimorphic shrub: anomalous female investment in Gynatrix pulchella? J Ecology. 94(6), 1261–1271 (2006).

[b24] Zhang C. Y., Wang J., Zhao X. H., Xia F. C. & Gadow K. V. Sexual dimorphism in reproductive and vegetative allometry for two dioecious Rhamnus plants in north-eastern China. Eur J For Res. 131, 1287–1296 (2012).

[b25] Nicotra A. B. Sexually dimorphic growth in the dioecious tropical shrub Siparuna grandiflora. Funct Ecol. 13, 322–331 (1999).

[b26] Massei G., Watkins R. & Hartley S. E. Sex-related growth and secondary compounds in Juniperus oxycedrus macrocarpa. Acta Oeco. 29(2), 135–140 (2006).

[b27] Bloom A. J., Chapin F. S. & Mooney H. A. Resource limitation in plants: an economic strategy. Ann Rev Ecol Syst. 16, 363–392 (1985).

[b28] Laure G. P., Salager J. L., Martine H. M. & Jacques R. Carbon allocation to volatiles and other reproductive components in male Ficus Carica (Moraceae). Am J Bot. 88, 2214–2220 (2001). 21669654

[b29] Harper J. L. The value of a leaf. Oecologia. 80, 53–58 (1989). 23494345 10.1007/BF00789931

[b30] Wilson P. J., Thompson K. & Hodgson J. G. Specific leaf area and leaf dry matter content as alternative predictors of plant strategies. New Phytol. 143(1), 155–162 (1999).

[b31] Arredondo J. T. & Sehnyder H. Components of leaf elongation rate and their relationship to specific leaf area in contrasting grasses. New Phytol. 158, 305–314 (2003).

[b32] Díaz-Barradas M. C., Zunzunegui M., Collantes M., Álvarez-Cansino L. & Novo F. G. Gender-related traits in the dioecious shrub *Empetrum rubrum* in two plant communities in the Magellanic steppe. Acta Oecol. 60, 40–48 (2014).

[b33] Makhlouf-Gafsi I., Mokni-Ghribi A., Bchir B., Attia H., Blecker C. & Besbes S. Physico-chemical properties and amino acid profiles of sap from Tunisian date palm. Sci Agr. 73(1), 85–90 (2016).

[b34] Sterner R. W. & Elser J. J. Ecological stoichiometry: the biology of elements from molecules to the biosphere. Princeton University Press (2002).

[b35] Gehring J. L. & Monson R. K. Sexual differences in gas exchange and response to environmental stress in dioecious Silene latifolia (Caryophyllaceae). Am J Bot. 166–174 (1994).

[b36] Che-Castaldo C., Crisafulli C. M., Bishop J. G. & Fagan W. F. What causes female bias in the secondary sex ratios of the dioecious woody shrub Salix sitchensis colonizing a primary successional landscape? Am J Bot. 102(8), 1309–1322 (2015). 26290554 10.3732/ajb.1500143

[b37] Wallace C. S. & Rundel P. W. Sexual dimorphism and resource allocation in male and female shrubs of *Simmondsia chinensis*. Oecologia. 44(1), 34–39 (1979). 10.1007/BF00346394 28310460

[b38] Zhang S., Jiang H., Zhao H., Korpelainen H. & Li C. Sexually different physiological responses of Populus cathayana to nitrogen and phosphorus deficiencies. Tree Physiol. tpu025 (2014). 10.1093/treephys/tpu025 24739232

[b39] Makino A. & Osmond B. Effects of nitrogen nutrition on nitrogen partitioning between chloroplast and mitoehondria in pea and wheat. Plant Physiol. 96, 355–362 (1991). 16668193 10.1104/pp.96.2.355 PMC1080777

[b40] Walcroft A. S., Whitehead D., Silvester W. B. & Kelliher F. M. The response of photosynthetic model parameters to temperature and nitrogen concentration in Pinus radiata D. Don. Plant Cell Environ. 20(11), 1338–1348 (1997).

[b41] Bourdeau P. F. Photosynthetic and respiratory rates in leaves of male and female quaking aspents. Forest Sci. 4, 331–334 (1958).

[b42] Laporte M. M. & Delph L. F. Sex-specific physiology and source-sink relations in the dioecious plant *Silene latifolia*. Oecologia. 106, 63–72 (1996). 10.1007/BF00334408 28307158

[b43] Marshall J. D., Dawson T. E. & Ehleringer J. R. Gender-related differences in gas exchange are not related to host quality in the xylem-tapping mistletoe, *Phoradendron juniperinum* (Viscaeae). Am J Bot. 35, 557–567 (1993).

[b44] Wang X. Z. & Gurtis P. S. Gender-specific response of *Populus tremuloides* to atmospheric CO_2_ enrichment. New Phytol. 150, 657–684 (2001).

[b45] Tolvanen A. & Henry G. H. Responses of carbon and nitrogen concentrations in high arctic plants to experimental warming. Can J Bot. 79(6), 711–718 (2001).

[b46] Demars B. O. & Edwards A. C. Tissue nutrient concentrations in freshwater aquatic macrophytes: high inter‐taxon differences and low phenotypic response to nutrient supply. Freshwater Biol. 52(11), 2073–2086 (2007).

[b47] Cornelissen J. H. C., Lavorel S., Gamier E. . A handbook of protocols for standardised and easy measurement of plant functional traits worldwide[J], Aust J Bot. 51(4), 335–380 (2003).

[b48] Tessier J. T. & Raynal D. J. Use of nitrogen to phosphorus ratios in plant tissue as an indicator of nutrient limitation and nitrogen saturation. J Appl Eco. 40(3), 523–534 (2003).

[b49] Vanni M. J., Flecker A. S., Hood J. M. & Headworth J. L. Stoichiometry of nutrient recycling by vertebrates in a tropical stream: linking species identity and ecosystem processes. Ecol Lett. 5(2), 285–293 (2002).

[b50] Zhang C., Zhao X., Gao L. & Gadow K. V. Gender, neighboring competition and habitat effects on the stem growth in dioecious *Fraxinus mandshurica* trees in a northern temperate forest. *Ann* Forest Sci. 66, 8–812 (2009).

[b51] Ågren G. I. The C: N: P stoichiometry of autotrophs–theory and observations. Ecol Lett. 7(3), 185–191 (2004).

[b52] Gernusak L. A., Winter K. & Turner B. L. Leaf nitrogen to phosphotus ratios of tropical trees: experimental assessment of physiological and environmental controls. New Phytol. 185, 770–779 (2009). 19968799 10.1111/j.1469-8137.2009.03106.x

[b53] Mate R., Johansson T. & Sitoe A. Biomass equations for tropical forest tree species in Mozambique. Forests. 5(3), 535–556 (2014).

[b54] Garnier E., Shipley B., Roumet C. & Laurent G. A standardized protocol for the determination of specific leaf area and leaf dry matter content. Funct Ecol. 15(5), 688–695 (2001).

[b55] Bao S. D. Analysis method of soil agricultural chemistry. Third edition. Chinese Agricultural Press. 135–155 (Beijing, 2000).

[b56] Warton D. J., Wright I. J., Falster D. S. & Westoby M. Bivariate line-fitting methods for allometry. Biol Rev. 81, 259–291 (2006). 16573844 10.1017/S1464793106007007

[b57] Fanin N., Fromin N., Buatois B. & Hättenschwiler S. An experimental test of the hypothesis of non‐homeostatic consumer stoichiometry in a plant litter–microbe system. Ecol lett. 16(6), 764–772 (2013). 23521784 10.1111/ele.12108

[b58] Sterner R. W., Andersen T., Elser J. J., Hessen D. O., Hood J. M., McCauley E. & Urabe J. Scale-dependent carbon: nitrogen: phosphorus seston stoichiometry in marine and freshwaters. Limnol Oceanogr. 53(3), 1169 (2008).

[b59] McArdle Brian, H. Lines, models, and errors: regression in the field. Limnol Oceanogr. 48(3), 1363–1366 (2003).

